# Hypoxanthine Induces Muscular ATP Depletion and Fatigue via UCP2

**DOI:** 10.3389/fphys.2021.647743

**Published:** 2021-03-03

**Authors:** Cong Yin, Zewei Ma, Fan Li, Chen Duan, Yexian Yuan, Canjun Zhu, Lina Wang, Xiaotong Zhu, Songbo Wang, Ping Gao, Gang Shu, Huihua Zhang, Qingyan Jiang

**Affiliations:** ^1^Guangdong Laboratory of Lingnan Modern Agriculture, National Engineering Research Center for Breeding Swine Industry and Guangdong Province Key Laboratory of Animal Nutritional Regulation, College of Animal Science, South China Agricultural University, Guangzhou, China; ^2^College of Life and Science, Foshan University, Foshan, China

**Keywords:** hypoxanthine, exercise, skeletal muscle, UCP2, ATP, fatigue

## Abstract

Hypoxanthine (Hx), an intermediate metabolite of the purine metabolism pathway which is dramatically increased in blood and skeletal muscle during muscle contraction and metabolism, is characterized as a marker of exercise exhaustion. However, the physiological effects of Hx on skeletal muscle remain unknown. Herein, we demonstrate that chronic treatment with Hx through dietary supplementation resulted in skeletal muscle fatigue and impaired the exercise performance of mice without affecting their growth and skeletal muscle development. Hx increased the uncoupling protein 2 (UCP2) expression in the skeletal muscle, which led to decreased energy substrate storage and enhanced glycolysis. These effects could also be verified in acute treatment with Hx through intraperitoneal injection. In addition, muscular specifically knockout of UCP2 through intra-muscle tissue injection of adenovirus-associated virus reversed the effects of Hx. In conclusion, we identified a novel role of Hx in the skeletal muscular fatigue mediated by UCP2-dependent mitochondrial uncoupling. This finding may shed light on the pathological mechanism of clinical muscle dysfunctions due to abnormal metabolism, such as muscle fatigue and weakness.

## Introduction

Skeletal muscle, the largest metabolic tissue of the body, is the major source of many metabolites that are released primarily during muscle contraction but also in the resting state (Pedersen and Febbraio, 2012; Seiler et al., 2015; Hoffmann and Weigert, 2017). These intermediates are characterized as myometabolites because of the important determinant of their levels in the circulation of muscle. Besides their role as energy substrates for many other metabolic pathways, myometabolites have received considerable attention due to their potential positive signaling roles in maintaining physical homeostasis and pathological progression, including exercise adaptation, body weight regulation (Hoffmann and Weigert, 2017), inflammation and immunological disease (Korotkova and Lundberg, 2014), insulin sensitivity and glucose homeostasis (Rai and Demontis, 2016), cancer and tumor formation (Yang et al., 2017), and cognitive function (Moon and van Praag, 2014). However, most studies on myometabolites are focused on their beneficial functions, while the detrimental roles are poorly understood. Studies on the detrimental myometabolites are mostly about lactate, which is produced and accumulated during muscle contraction, and could cause muscle fatigue and soreness. These detrimental myometabolites should also be paid attention, so that we could fully understand the physiological roles of them.

Hypoxanthine (Hx), an intermediate metabolite of purine nucleotides, is also an important myometabolite that mainly distributed in muscular tissue (Bone et al., 2015). During exercise or muscle contraction, Adenosine triphosphate (ATP) is catabolized to release high-energy phosphate for energy demand. Meanwhile, it produces numerous intermediate metabolites in this metabolic process, such as: adenosine monophosphate (AMP), inosine monophosphate (IMP), adenosine, inosine, and Hx (Zielinski et al., 2013a; Hoffmann and Weigert, 2017). Many studies on energy metabolism of exercise have identified the elevation of blood Hx level, which could be 2-10 times of the resting state in response to different exercise intensity (Zielinski et al., 2009, 2013a,b; Zieliński and Kusy, 2015). So Hx is also characterized as a marker of exercise exhaustion or fatigue. Moreover, Hx is involved in various physiological and pathological processes, for instance, cholesterol metabolism and atherosclerosis (Ryu et al., 2016), cell apoptosis (Kim et al., 2017), neural signaling presentation (Zamzow et al., 2008; Biasibetti et al., 2017), intestinal barrier protection (Lee et al., 2018), and embryonic development (Dienhart et al., 1997; Ma et al., 2003). However, the physiological roles of Hx in the skeletal muscle metabolism remain unknown.

The regulation of skeletal muscle metabolism involves changes in muscle mass, myofiber composition, and mitochondrial properties associated with appropriate metabolic modifications. Slow and fast myofibers in skeletal muscle exhibit different oxidative and glycolytic metabolic capacities, and can be converted from one fiber type to another in response to the metabolic demand (Frontera and Ochala, 2015). As the core element of energy metabolism, mitochondria determine the utilization of metabolic substrates and the transformation of metabolic pathways. Uncoupling proteins (U), located in the inner mitochondrial membrane, can dissipate the proton electrochemical gradient generated during electron transport (Bouillaud et al., 2016). Consequently, UCPs eliminate the reactive oxygen species (ROS) and protect the cell from oxidative stress. On the other hand, UCPs uncouple mitochondrial respiration from ATP formation, and the direct consequence of this process is a decrease in the oxidative phosphorylation efficacy.

In this study, we investigated the role of Hx in muscle physiological function. We found that Hx causes muscular weakness of mice both through chronic treatment (dietary supplement) and acute treatment (intraperitoneal injection). Hx induced the muscular ATP deficiency and enhanced glycolysis that involves the uncoupling protein 2 (UCP2). The expression of UCP2 was elevated with Hx-treated. Muscular specific knockout of UCP2 through adeno-associated viral vector injection blocked Hx-induced ATP deficiency and glycolysis enhancement, which rescued muscular weakness. These findings might be relevant to the mechanism of muscle fatigue and weakness.

## Materials and Methods

### Animal Experiments

Animal experiments were approved by the College of Animal Science, South China Agricultural University. Wild-type C57BL/6J male mice were maintained in accordance with The Instructive Notions with Respect to Caring for Laboratory Animals (Ministry of Science and Technology, Beijing, China). Mice were housed in individual cages with a constant temperature of 25 ± 1°C, 55% relative humidity, and 12 h light/dark cycle.

For chronic Hx-treated experiment, 24 male mice of 7 weeks old were randomly divided into three groups according to body weight (8 mice per group), the mice were given *ad libitum* access to a standard chow diet with or without Hx (10 mg/g or 20 mg/g) (Sango Biotech, China, purity ≥ 98.0%) and water. The body weight and food intake were monitored for 4 weeks, and then the body composition was measured by a nuclear magnetic resonance system (Body Composition Analyzer MiniQMR23-060H-I, Niumag, China). Subsequently, the exercise capacity was measured. The mice were sacrificed 1 week after exercise measurement. Blood was collected for separation of serum. Different muscles in the hind limb (gastrocnemius, GAS; tibialis anterior, TA; soleus, SOL; and extensor digitorum longus, EDL) were separated for the subsequent detection and analysis.

For acute Hx-treated experiment, 16 male mice of 10 weeks old were randomly divided into two groups according to body weight (8 mice per group), the mice were given an intraperitoneal (i.p.) injection of Hx (30 mg/kg body weight) or saline. 30 min after injection, the mice performed exercise capacity assessment, energy metabolism measurements, or were sacrificed for collecting blood and muscle samples.

### Adeno-Associated Viral Vector 9 (AAV9) Injection

Thirty-two male mice of 7 weeks old were randomly divided into four groups according to body weight (8 mice per group). AAV-GFP-*Cas9* combined with AAV9-GFP-*Ucp2*-sgRNA (abm, Canada) was injected into the GAS muscle of both hind limbs of the mice in two groups for knockout of *Ucp2* (m*UCP2*KO groups). AAV-GFP-*Cas9* combined with AAV9-GFP-*scramble*-sgRNA (abm, Canada) was injected into the GAS muscle of both hind limbs of the mice in the other two groups as negative control (control groups). Titer of the mixed virus was at least 10^11^. The validated sgRNA sequence for knockout of *Ucp2* is (target 1: TGGTCTTTCAGATCCAAGGG, target 2: GCGCCCAGTACCGTGGCGTT, and target 3: TCCTAACCATGGTGCGCACT) (abm, Canada). 3 weeks after infection, the mice took part in the acute Hx-treated experiment as described above. In brief, the mice in the control groups or m*UCP2*KO groups were given an i.p. injection of Hx or saline. 30 min after injection, the mice performed exercise capacity assessment or were sacrificed.

### Exercise Capacity Assessment

The exhaustion test was performed on a rodent treadmill (47303, Ugo, Italy) according to the following protocol: 8 m/min for 5 min, 10 m/min for 5 min, subsequently, velocity was increased by 2 m/min every 20 min until mice reached exhaustion at an inclination of 10°. Exhaustion was defined as the point at which the mice spent more than 5 s on the electric shocker without attempting to resume running. Adaption training session was performed three times before the experiment with 10 m/min for 10 min (10° incline) for each time.

For the grip strength test, muscle strength was measured by a grip test meter (BIO-GS3, Bioseb, France). The mice were held on to a metal grid with four paws and were gently pulled backward by the tail until the animals could no longer hold the grid. Each mouse was given eight trials, and the average values were used to represent the muscle grip strength of an individual mouse.

For weights lifting test, which was also performed to measure muscle strength that referred to research of Lahiri et al. (2019). Briefly, each mouse was grasped in the middle of the tail, and then grasped the first weight (26 g). After the mouse grasped the weight, the mouse was slowly raised until the weight was completely suspended. The criterion was met if the mouse could hold the weight for 3 s. If the mouse cannot hold for 3 s, rest for 10 s and repeat for five times maximum. If it successfully held the weight for 3 s, then it was allowed to progress to the next heaviest weight. The apparatus comprised six weights, weighing 26, 33, 44, 63, 82, 100, and 120 g respectively. 26 g is counted as 1 point, 33 g is 2 points, 44 g is 3 points, 63 g is 4 points, 82 g is 5 points, 100 g is 6 points, and 120 g is 7 points. The total score of each mouse is equal to the cumulative score of all weights that can be lifted for 3 s.

### Biochemical Assays

Serum glucose, non-esterified fatty acid (NEFA), aspartate aminotransferase (AST), Alanine transaminase (ALT), and blood urea nitrogen (BUN), GAS muscle glycogen and lactate were measured by using the biochemistry detection kits (Nanjing Jiancheng Bioengineering Institute, China). The ATP contents in the GAS muscle tissues were measured using the ATP Assay Kit (S0026, Beyotime, China). The ROS contents of the GAS muscle tissues were measured using a tissue ROS Assay Kit (BB-470515-1, BestBio, China). All assays were performed according to the manufacturer’s instructions.

### Energy Metabolism Measurements

For metabolic studies *in vivo*, mice were housed individually in the Promethion Metabolic Screening Systems (Sable Systems International, North Las Vegas, NV, United States) with free access to corresponding food and water. After acclimatization to the systems for 24 h, energy expenditure (EE), respiration exchange ratio (V_*CO*__2_/V_*O*__2_), and locomotor activity were monitored for the following 48 h. Data were collected and analyzed by MetaScreen-Data Collection Software (V2.3.17) and Expedata-P Data Analysis Software (V1.9.17), respectively.

### Immunofluorescence

The GAS muscles were separated as soon as the mice were sacrificed and frozen in liquid nitrogen, transverse 10-μm sections were generated by using the freezing microtome (CM1950, Leica, Germany). Sections were fixed in 4% paraformaldehyde, permeabilized in 0.5% Triton-X 100, and blocked in 5% BSA with 10% goat serum. Muscle slices were incubated with the primary antibodies overnight at 4°C, washed and incubated with the secondary AlexaFluor-conjugated antibodies for 1 h at room temperature. Slices were imaged using a Nikon Eclipse Ti-s microscope. Images were processed and analyzed with ImageJ (NIH). The following antibodies were used: rabbit anti-laminin antibody (1:500, A19970, ABclonal, China), mouse anti-MyHC I antibody (1:500, BA-D5-s, DSHB, United States), and mouse anti-MyHC IIb antibody (1:50, BF-F3, DSHB, United States).

### RNA Isolation and Quantitative RT-PCR (qRT-PCR)

Total RNA of the GAS muscle tissues was extracted using the Hipure Universal RNA Mini kit (R4130-02, Magen, China) according to the manufacturer’s instructions. A total 2 μg of RNA was converted into cDNA with random primers using the M-MLV enzyme (Promega, United States). qRT-PCR was performed with a QuantStudio 3 Flex Real-Time PCR system (7300HT, Applied Biosystems, United States) using SYBR Green master mix as per the manufacturer’s instructions (Q711, Vazyme, China). Normalized mRNA expression was calculated by using the 2^–ΔΔ*CT*^ method, β*-actin* was used as the reference gene. The list of primer sequences is presented in Table 1.

**TABLE 1:** Primer sequences used for qRT-PCR.

Gene	Forward primer sequence (5′–3′)	Reverse primer sequence (5′–3′)
*MyHC I*	ACCAGGCCCTTTGACCTCAA GAAA	TCTTGTCGAACTTGGGTGGGT TCT
*MyHC IIa*	AGTCCCAGGTCAACAAGCTG	TTTCTCCTGTCACCTCTCAACA
*MyHC IIb*	AGTCCCAGGTCAACAAGCTG	TTTCTCCTGTCACCTCTCAACA
*Hk2*	GGACGGAATTCAGAAGGCCT	TCCTCGCCCTTGTTCTCTTT
*Eno2*	GAAGGAAGCCATCGACAAGG	TGGTCCCCAGTGATGTATCG
*Gapdh*	AGTGTTTCCTCGTCCCGTAG	GCCGTGAGTGGAGTCATACT
*Ldha*	GTCCAGCGAAACGTGAACAT	GCCACTGATTTTCCAAGCCA
*Gck*	GAAAGTACATGGGCGAGCTG	AAACGGGTCTCAAAAGCACC
*Pkm*	CTTCATTCAGACCCAGCAGC	CCGAGCCACATTCATTCCAG
*Cpt1b*	CTGGGATGCGTGTAGTGTTG	TCATGTATCGCCGCAAACTG
*Crat*	GGGCAGTTCTTCTAAGGCAG	TATGCCGCTATGTCTGGTCT
*Acadm*	ACACCCATACGCCAACTCTT	AGTACCCGTTCCCTCTCATC
*Acads*	GTAGGCCAGGTAATCCAAGCC	TGGCGACGGTTACACACTG
*Nrf1*	CCACGTTGGATGAGTACACG	CTGAGCCTGGGTCATTTTGT
*Tfam*	AGATATGGGTGTGGCCCTTG	AAAGCCTGGCAGCTTCTTTG
*Cox6a1*	TGCTCAACGTGTTCCTCAAG	TAAGGGTCCAAAACCAGTGC
*Atp5b*	GAGGGATTACCACCCATCCT	CATGATTCTGCCCAAGGTCT
*Ndufa6*	GTCACAGACCCCAGAGTGGT	TAACATGCACCTTCCCATCA
*Ucp2*	TCCTGCACCCCGATTTACTT	CTTTATGGGTGAAGGCTGGC
*Ucp3*	CCGTTTTGAACAAGGCAAGC	GTTTCTCGTGTCAGCAGCAG
*Gss*	GTTGCTGTGGTGTACTTCCG	CACCTTCTTAGTCCCAGCCA
*Gpx4*	TTGATAAGAACGGCTGCGTG	AGACCTTCATGAGTGCCGG
β*-actin*	TAGGCGGACTGTTACTGAGC	AATCCTGAGTCAAAAGCGCC

*MyHC I, myosin heavy chain I; MyHC IIa, myosin heavy chain IIa; MyHC IIb, myosin heavy chain IIb; Hk2, hexokinase 2; Eno2, enolase 2; Gapdh, glyceraldehyde-3-phosphate dehydrogenase; Ldha, lactate dehydrogenase A; Gck, glucokinase; Pkm, pyruvate kinase M1/2; Cpt1b, carnitine palmitoyltransferase 1B; Crat, carnitine O-acetyltransferase; Acadm, acyl-CoA dehydrogenase medium chain; Acads, acyl-CoA dehydrogenase short chain; Nrf1, nuclear respiratory factor 1; Tfam, mitochondrial transcription factor A; Cox6a1, cytochrome c oxidase subunit 6a1; Atp5b, ATP synthase, H^+^ transporting mitochondrial F1 complex, beta subunit; Ndufa6, NADH: ubiquinone oxidoreductase subunit A6; Ucp2, uncoupling protein 2; Ucp3, uncoupling protein 3; Gss, glutathione synthetase; Gpx4, glutathione peroxidase 4.*

### Protein Extraction and Western Blot

The GAS muscle tissues lysates were obtained using RIPA lysis buffer freshly supplemented with proteinase inhibitor (BB-3101-2, BestBio, China). The protein samples were separated by SDS-PAGE and transferred onto PVDF membranes (Merck Millipore, Germany), after blocking in 5% defatted milk dissolved in TBST. The membranes were incubated with primary antibodies overnight at 4°C and with HRP-conjugated secondary antibody for 1 h at room temperature. The signal was visualized by using ECL substrate (Merck Millipore, Germany). The protein band intensity was quantified by ImageJ (NIH). The following antibodies were used: rabbit anti-UCP2 antibody (1:1500, 162448, ZENBIO, China), rabbit anti-UCP3 antibody (1:1500, 162449, ZENBIO, China), mouse anti-MyHC I antibody (1:1000, BA-D5-s, DSHB, United States), mouse anti-MyHC IIb antibody (1:1000, BF-F3, DSHB, United States), and rabbit anti-β-actin antibody (1:5000; AP0060, Bioworld, China).

### Statistical Analysis

All data were presented as means ± standard error of the mean (SEM) for experiments, including the numbers of mice in each group as indicated. Unpaired Student’s *t*-test or one-way ANOVA analysis followed by *post hoc* Tukey’s test was performed for statistical significance comparison by using the GraphPad Prism 8 software (GraphPad).

## Results

### Chronic Hx-Treated Induces Exercise Weakness of Mice

**FIGURE 1:**
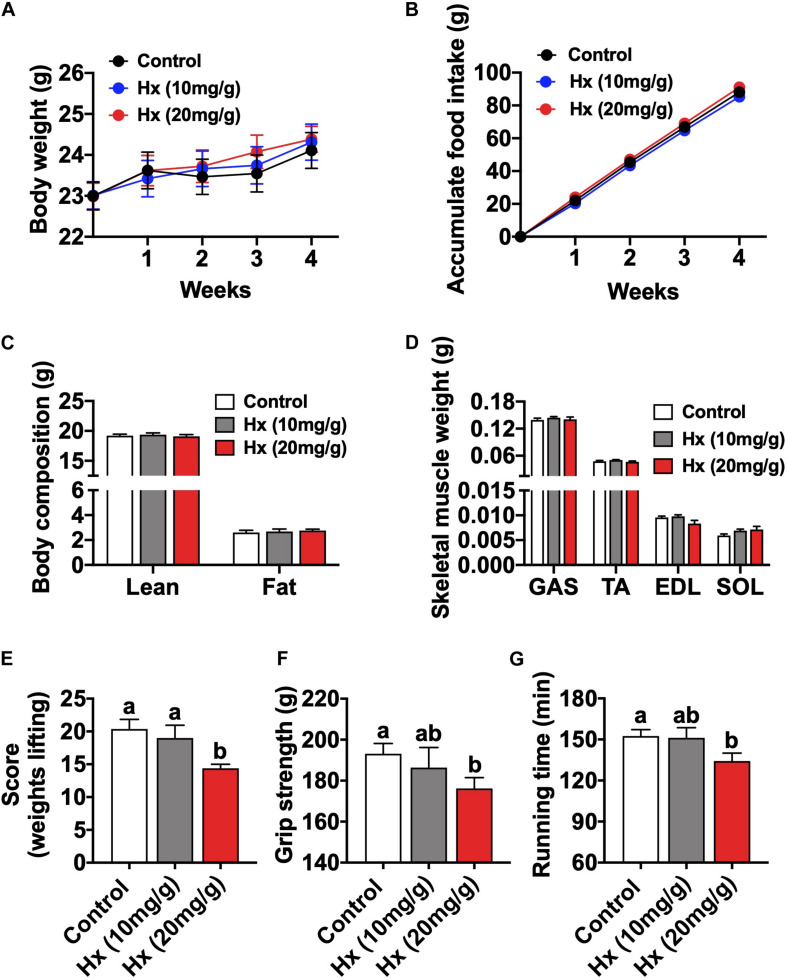
Chronic Hx-treated induces muscular fatigue. **(A)** Body weight after 4 weeks Hx treatment (*n* = 8 mice per group). **(B)** Cumulative food intake after 4 weeks Hx treatment (*n* = 8 mice per group). **(C)** Body composition of mice after 4 weeks Hx treatment (*n* = 8 mice per group). **(D)** Skeletal muscle weight (GAS; tibialis anterior, TA; soleus, SOL; and extensor digitorum longus, EDL) (*n* = 8 mice per group). **(E)** Weights lifting score after Hx treatment (*n* = 8 mice per group). **(F)** Grip strength after Hx treatment (*n* = 8 mice per group). **(G)** Treadmill running time after Hx treatment (*n* = 8 mice per group). Values are presented as means ± SEM, different letters between bars mean *P* ≤ 0.05 in one-way ANOVA analysis followed by *post hoc* Tukey’s tests.

To investigate the physiological roles of Hx in the skeletal muscle and exercise, mice were treated with Hx through dietary supplement (0, 10, or 20 mg/g in diet). After 4 weeks of feeding, the body weight (Figure 1A) and accumulated food intake (Figure 1B) of the Hx-treated mice (both the 10 mg/g group and 20 mg/g group) were comparable to the control, and so were the lean mass, fat mass, and skeletal muscle weight (GAS, TA, EDL, and SOL) (Figures 1C,D). In addition, there was also no difference in mice growth and development between the 10 and 20 mg/g Hx-treated group (Figures 1A–D). Subsequently, the mice performed exercise capacity assessments including the strength test (weight lifting test score or grip strength) and the endurance test (treadmill running). We found that Hx induced exercise weakness depended on the dosage. The mice in the 20 mg/g Hx treated group showed lower weight lifting test scores (*P* ≤ 0.05, Figure 1E), weaker muscle grip strength (*P* ≤ 0.05, Figure 1F), and lower running endurance (*P* ≤ 0.05, Figure 1G). There was no significant change in 10 mg/g Hx treated group compared to the control, although the weight lifting score and grip strength were slightly reduced after treatment with 10 mg/g Hx. Moreover, the weight lifting scores were significantly decreased in the 20 mg/g Hx-treated group compared to the 10 mg/g Hx-treated group (*P* ≤ 0.05, Figure 1E), while there was no significant difference in the grip strength and running time (Figures 1F,G). Hence, we chose the additive concentration of 20 mg/g for the subsequent experiments.

**FIGURE 2:**
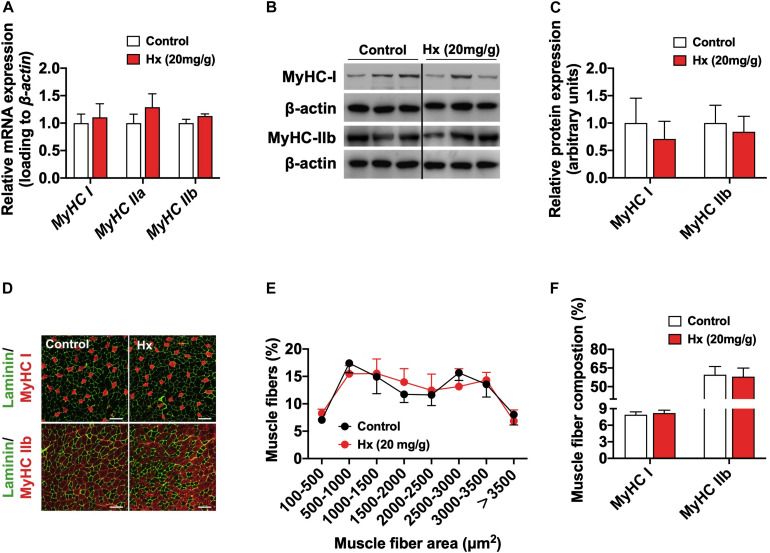
Chronic Hx-treated does not alter muscle fiber area and fiber type composition. **(A)** mRNA expression of genes related to the myofiber types (*n* = 8–10 mice per group). **(B,C)** Western blot (B) and quantification **(C)** of MyHC I and MyHC IIb content in the GAS muscle (*n* = 6 per group). Dividing lines indicate spliced bands from the same gel. **(D)** Immunofluorescence staining of Laminin (green), MyHC I (upper, red), and MyHC IIb (lower, red) of GAS muscle section (bars = 50 μm). **(E,F)** Statistical analysis of muscle fiber area **(E)** and fiber type composition **(F)** according to immunofluorescence staining of muscle sections (*n* = 3 mice per group). β-actin was used as loading control both in qRT-PCR and western blot. Values are presented as means ± SEM.

Muscular plasticity is characterized as the muscle fiber development and myofiber type transformation in response to different internal or external stimuli (Allen et al., 2008). Then, we measured the muscle fiber size and fiber type composition. The mRNA and protein levels of myofiber type markers were comparable between the Hx-treated group and the control (Figures 2A–C). The immunofluorescence staining of Laminin (reflecting the boundary of myofiber) and myofiber type markers of muscle sections (slow-oxidative type, MyHC I; and fast-glycolytic type, MyHC IIb) also showed no differences in the myofiber type composition and fiber size between the two groups (Figures 2D–F). All these results show that chronic Hx intake induces exercise weakness of mice, without affecting the growth performance, myofiber size and fiber type composition.

### Hypoxanthine Alters Muscular Energy Metabolism and Reduces ATP Content

**FIGURE 3:**
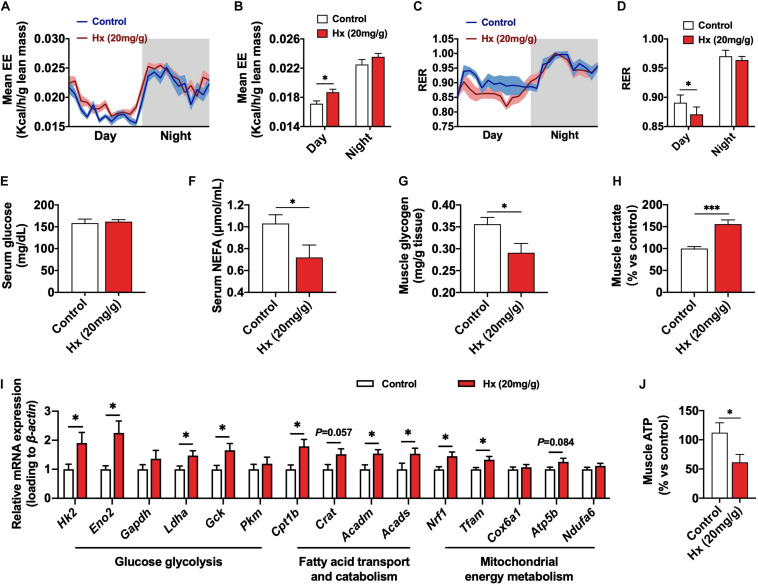
Effects of Hx on the skeletal muscle energy metabolism. **(A)** EE of mice after 4 weeks Hx treatment. Grey areas indicate dark periods (*n* = 8 mice per group). **(B)** Statistics of mean EE values of mice in night or dark cycle after 4 weeks Hx treatment (*n* = 8 mice per group). **(C)** RER of mice after 4 weeks Hx treatment. Gray areas indicate dark periods (*n* = 8 mice per group). **(D)** Statistics of mean RER values of mice in night or dark cycle after 4 weeks Hx treatment (*n* = 8 mice per group). **(E–H)** Serum glucose **(E)** and NFFA **(F)** levels; GAS muscle glycogen **(G)**, and lactate **(H)** content of mice after 4 weeks Hx treatment (*n* = 8 mice per group). **(I)** mRNA expression of genes related to the glucose glycolysis, fatty acid transport and catabolism, and mitochondrial energy metabolism of GAS muscle after 4 weeks Hx treatment (*n* = 7–8 mice per group). **(J)** GAS muscle ATP content of mice after 4 weeks Hx treatment (*n* = 8 mice per group). β*-actin* was used as loading control in the qRT-PCR. Values are presented as means ± SEM, ^∗^*P* ≤ 0.05 and ^∗∗∗^*P* ≤ 0.001 according to non-paired Student’s *t*-test between individual groups.

We eliminated the influence of muscle fiber structure and composition on Hx-induced exercise fatigue. Consequently, we considered the role of skeletal muscle metabolism, which is also another key determinant in muscle function. Firstly, we measured the global energy metabolism of mice. Figure 3A showed the dynamic change of average EE per hour in the whole day. The EE was elevated in the Hx-treated group and was significantly different in the light cycle (*P* ≤ 0.05, 3B), while the locomotor activity was similar between the two groups (Supplementary Figure 1), indicating that the enhanced metabolism was not due to the alteration of physical activity. Moreover, the respiratory exchange ratio (RER) was significantly lower in Hx-treated mice compare to the control (*P* ≤ 0.05, Figures 3C,D), suggesting that Hx induced a metabolic tendency to use lipid as an energy substrate. Then, we investigated the main biochemical parameters related to muscle contraction metabolism of mice in circulation blood and muscle tissue. There was no difference in serum glucose between Hx-treated mice and controls (Figure 3E), but we found the significant decrease of NFFA of serum (*P* ≤ 0.05, Figure 3F), which might show the increased mobilization of lipid substrate, which was consistent with the RER decreasing with the Hx-treated group. Moreover, decreased GAS muscle glycogen (*P* ≤ 0.05, Figure 3G) and increased muscle lactate (*P* ≤ 0.001, Figure 3H) was in response to Hx-treatment. Based on the alteration of these metabolic indicators, we further measured the mRNA expression of genes related to glucose glycolysis (*Hk2*, *Eno2*, *Gapdh*, *Ldha*, *Gck*, and *Pkm*), fatty acid transport (*Cpt1b* and *Crat*) and catabolism (*Acadm* and *Acads*), and mitochondrial biogenesis (*Nrf1* and *Tfam*) and oxidative phosphorylation (*Cox6a1*, *Atp5b*, and *Ndufa6*). It showed the significant elevation of these pathways or processes (*P* ≤ 0.05, Figure 3I), which was consistent with the previous results. ATP is the direct energy supply substance for muscle contraction. Interestingly, the muscular ATP was lower in the administration of Hx compared with the controls (*P* ≤ 0.05, Figure 3J), this might be the key point in Hx-induced muscular weakness. Altogether, these data indicate that Hx caused an increase in cellular energy metabolism, but depressed ATP production in skeletal muscle.

### Acute Hx-Treated Also Induces Muscular Metabolic Transformation and Weakness

**FIGURE 4:**
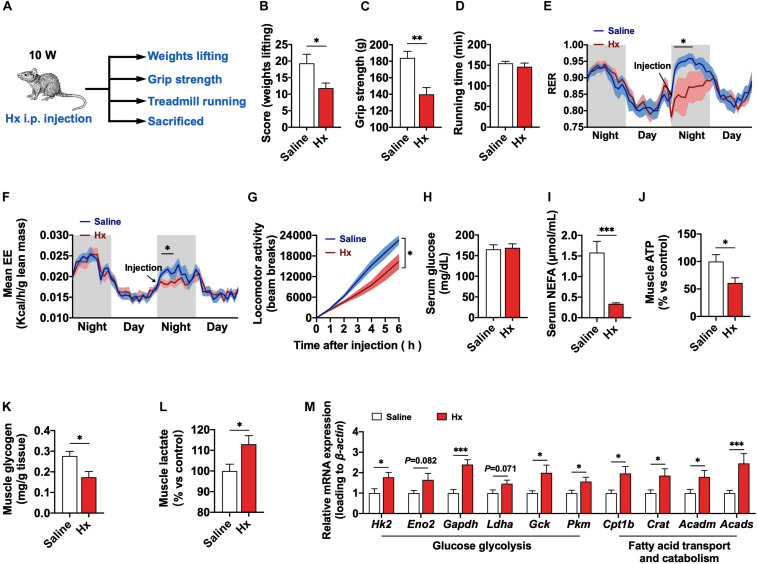
Acute effects of Hx on the exercise capacity and metabolism of mice. **(A)** Experimental scheme for the acute Hx treatment of mice. **(B)** Weights lifting score after acute Hx treatment (*n* = 8 mice per group). **(C)** Grip strength after acute Hx treatment (*n* = 8 mice per group). **(D)** Treadmill running time after acute Hx treatment (*n* = 8 mice per group). **(E,F)** RER and EE of mice. The arrow pointed to the time of acute i.p. injection. Gray area indicated dark period (*n* = 8 mice per group). **(G)** Accumulated locomotor activity (bean breaks) of mice after acute Hx treatment (*n* = 8 mice per group). **(H–L)** Serum glucose **(H)** and NEFA **(I)** levels; GAS muscle ATP **(J)**, glycogen **(K)**, and lactate **(L)** content of mice after acute Hx treatment (*n* = 7–8 mice per group). **(M)** mRNA expression of genes related to the glucose glycolysis and fatty acid transport and catabolism of GAS muscle after acute Hx treatment (*n* = 7–8 mice per group). β*-actin* was used as loading control in the qRT-PCR. Values are presented as means ± SEM; ^∗^*P* ≤ 0.05, ^∗∗^*P* ≤ 0.01, and ^∗∗∗^*P* ≤ 0.001 according to the non-paired Student’s *t*-test between individual groups.

Grip strength and weight lifting test both indicate the transient strength of muscle. Based on the effect of chronic Hx-treatment in skeletal muscle, we aimed to explore the acute effect of Hx in skeletal muscle. The mice were i.p. injected with Hx (30 mg/kg body weight) or saline, and then performed muscle grip test, weights lifting test, and treadmill running test, respectively (Figure 4A). Though treadmill running was not altered in Hx-treated mice compared with the control ones (Figure 4D). The weights lifting score of mice was significantly decreased after Hx-treatment (*P* ≤ 0.05, Figure 4B), as well as the grip strength (*P* ≤ 0.01, Figure 4C), which was consistent with the chronic treatment. The RER of mice was dramatically decreased within 6 h after Hx-treatment (*P* ≤ 0.05, Figure 4E), which was similar to the chronic effect. But contrary to the chronic-treated, Hx decreased the EE of mice within 3 h after i.p. injection (*P* ≤ 0.05, Figure 4F), which might be due to the reduced locomotor activity after Hx-treatment (*P* ≤ 0.05, Figure 4G). The reduced locomotor activity was not due to the acute toxicity of Hx, it was because that there was no difference in the serum biochemical indicators, ASL, ALT, and BUN, which are related to the acute toxicity, with Hx-treatment compared with the control (Supplementary Figures 2A–C). We also measured the biochemical targets in serum and muscle. Consistently, we found the invariable serum glucose (Figure 4H), decreased serum NEFA (*P* ≤ 0.001, Figure 4I), reduced muscular ATP (*P* ≤ 0.05, Figure 4J) and glycogen (*P* ≤ 0.05, Figure 4K), and elevated muscular lactate with Hx-treatment (*P* ≤ 0.05, Figure 4L). Moreover, the mRNA expression of glucose glycolysis (*Hk2*, *Eno2*, *Gapdh*, *Ldha*, *Gck*, and *Pkm*) or fatty acid transport and catabolism genes (*Cpt1b*, *Crat*, *Acadm*, and *Acads*) were significantly increased as well (*P* ≤ 0.05, Figure 4M). Together, these data show that acute Hx-treatment also induces muscular metabolic transformation and exercise weakness.

### Hypoxanthine Induces the Activation of UCP2 in Skeletal Muscle

**FIGURE 5:**
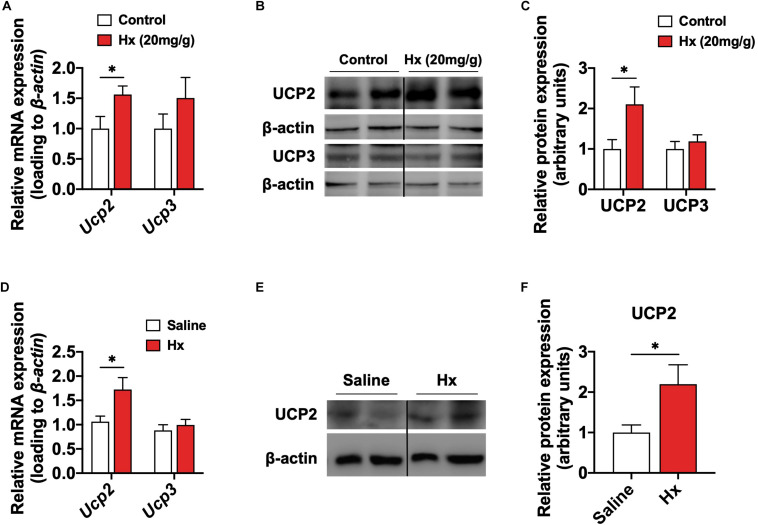
Hx induces the activation of UCP2 in skeletal muscle. **(A)** mRNA expression of *UCP2* and *UCP3* in the GAS muscle after 4 weeks Hx treatment (*n* = 8 mice per group). **(B,C)** Western blot **(B)** and quantification **(C)** of UCP2 and UCP3 content in the GAS muscle after 4 weeks Hx treatment (*n* = 6 per group). Dividing lines indicate spliced bands from the same gel. **(D)** mRNA expression of *UCP2* and *UCP3* in the GAS muscle after acute Hx treatment (*n* = 8 mice per group). **(E,F)** Western blot **(E)** and quantification **(F)** of UCP2 content in the GAS muscle after acute Hx treatment (*n* = 6 per group). Dividing lines indicate spliced bands from the same gel. β-actin was used as loading control both in qRT-PCR and western blot. Values are presented as means ± SEM. ^∗^*P* ≤ 0.05 according to the non-paired Student’s *t*-test between individual groups.

Generally, enhanced cellular energy metabolism should produce more energy substrate. But actually, the promotion of cellular energy metabolism induced by Hx was accompanied with a lack of ATP, that indicated that the metabolism was ineffective, caused by Hx. Hence, we focused on the UCPs that mediate the mitochondrial uncoupling process. It has reported that ATP production is suppressed by increased uncoupled respiration mediated through UCPs in any given mitochondria (Slocinska et al., 2016). Among the UCPs family, UCP1 is mainly expressed in brown adipose tissue, while the UCPs mainly expressed in skeletal muscle are UCP2 and UCP3 (Brand and Esteves, 2005). To validate the hypothesis related to UCPs, we detected the expressions of UCP2 and UCP3 in skeletal muscle. As expected, the mitochondrial UCP2, but not UCP3, was significantly increased with chronic Hx-treated both in the mRNA expression (*P* ≤ 0.05, Figure 5A) and protein levels (*P* ≤ 0.05, Figures 5B,C). Consistently, the mRNA expression (*P* ≤ 0.05, Figure 5D) and protein levels (*P* ≤ 0.05, Figures 5E,F) of UCP2 were also upregulated with acute Hx-treated. Enhanced mitochondrial metabolism could generate more oxidative free radicals that would be neutralized by UCPs and other peroxidase systems to maintain the intracellular redox homeostasis (Deyu et al., 2018). Moreover, ROS is also an important regulator of UCP2 (Mailloux and Harper, 2012). Herein, we observed no change in the muscle ROS content in the chronic Hx-treated group compared to the controls (Supplementary Figure 3A). However, Hx induced significantly increased mRNA expression of *glutathione synthetase* (*Gss) and glutathione peroxidase* (*Gpx4)*, which mediate the glutathione reduction system (*P* ≤ 0.05, Supplementary Figure 3B). These indicated the enhanced cleaning capacity in response to activated UCP2 and glutathione reduction system might have kept the ROS homeostasis. All these results suggest that UCP2 might be a potential target in Hx-induced muscular weakness.

### UCP2 Is Required for the Hx-Induced Muscular ATP Deficiency and Weakness

**FIGURE 6:**
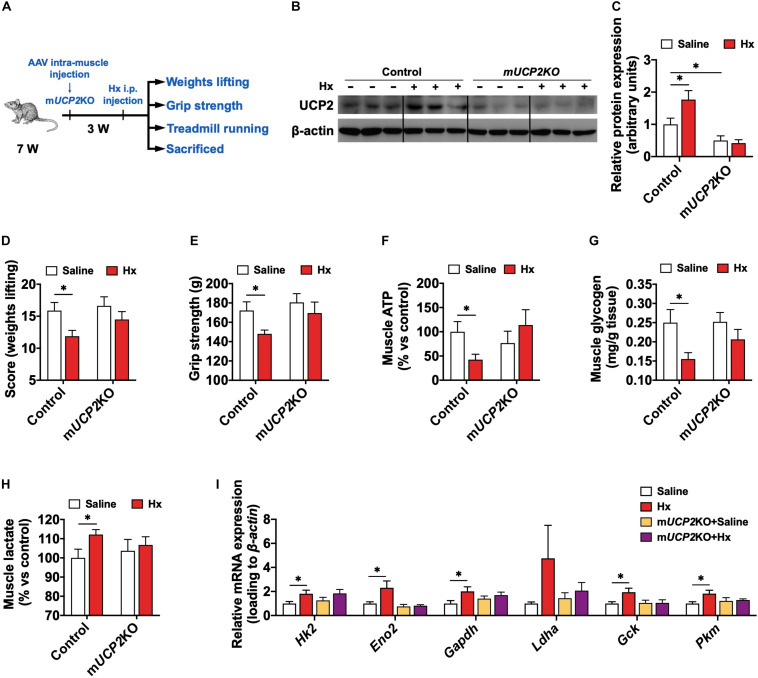
UCP2 is required for the Hx-induced muscular ATP deficiency and weakness. **(A)** Experimental scheme for the acute Hx treatment on m*UCP2*KO mice. **(B,C)** Western blot **(B)** and quantification **(C)** of UCP2 content in the GAS muscle after acute Hx treatment (*n* = 6 per group). Dividing lines indicate spliced bands from the same gel. **(D)** Weights lifting score after acute Hx treatment (*n* = 8 mice per group). **(E)** Grip strength after acute Hx treatment (*n* = 8 mice per group). **(F–H)** GAS muscle ATP **(F)**, glycogen **(G)**, and lactate **(H)** content of mice after acute Hx treatment (*n* = 7–8 mice per group). **(I)** mRNA expression of genes related to the glycolysis of GAS muscle after acute Hx treatment (*n* = 7–8 mice per group). β-actin was used as loading control both in qRT-PCR and western blot. Values are presented as means ± SEM; ^∗^*P* ≤ 0.05 according to the non-paired Student’s *t*-test between individual groups.

To further explore the functional importance of UCP2 in the Hx-induced muscular weakness, we used an intra-muscle tissue AAV injection strategy to knockout (KO) *Ucp2* in the GAS muscle through CRISPR-Cas9 system (Figure 6A). 3 weeks after injection, there was no difference in the body weight (Supplementary Figure 4A) and muscle mass (Supplementary Figure 4B) of mice between muscular *Ucp2* specifically KO (m*UCP2*KO) mice and the controls. Then, the mice were given i.p. injection of Hx (30 mg/kg body weight) or saline, and measured the weights lifting score, grip strength, or sacrificed for sample collection, respectively (Figure 6A). First, the m*UCP2*KO model was established successfully through the validation of reduced UCP2 levels in protein expression (*P* ≤ 0.05, Figures 6B,C). Acute Hx-treated induced significantly increased of UCP2 protein level in skeletal muscle (*P* ≤ 0.05, Figures 6B,C), and m*UCP2*KO blocked the Hx-induced UCP2 activation (Figures 6B,C). As expected, Hx induced significantly decreased weight lifting scores (*P* ≤ 0.05, Figure 6D), grip strength (*P* ≤ 0.05, Figure 6E), and muscle ATP content (*P* ≤ 0.05, Figure 6F) in mice, while m*UCP2*KO restored the exercise fatigability and ATP deficiency of mice (Figures 6D–F). The enhanced glycolysis in muscle which was presented by the reduced muscular glycogen (*P* ≤ 0.05, Figure 6G), increased muscular lactate content (*P* ≤ 0.05, Figure 6H), and elevation of mRNA expression of glycolysis genes (*Hk2*, *Eno2*, *Gapdh*, *Ldha*, *Gck*, and *Pkm*) with Hx-treatment (*P* ≤ 0.05, Figure 6I), was also reversed by m*UCP2*KO (Figures 6G–I). These results suggest the roles of UCP2 in Hx induced muscular glycolysis, ATP depletion, and weakness.

## Discussion

Herein, we showed that Hx impaired the exercise capacity of mice both with chronic and acute treatment. Mechanistically, Hx altered muscle energy metabolism, mainly reflected in enhanced glycolysis, accelerated energy substrate consumption and mobilization, and depressed ATP flowage. Otherwise, Hx induced UCP2 activation in skeletal muscle, which mediated the mitochondrial uncoupling process that blocked ATP production. Muscle specifically knocked out of UCP2 could block glycolysis elevation and rescue energy insufficiency and muscle fatigue with Hx-treatment. These results shed a new light on the physiological role of Hx in skeletal muscle metabolism and function.

Physical exercise is widely accepted to prevent several pathogenic conditions, such as hypertension, coronary heart disease (Downing and Balady, 2011), obesity (Heo et al., 2017), type 2 diabetes mellitus (Fan et al., 2017), age-related muscle wasting (sarcopenia) (Bowen et al., 2015), and cancer (Hojman et al., 2018), largely due to metabolic adaptations induced by contraction in skeletal muscle. Yet how exercise induces the effects remains to be fully understood. It is generally believed that a cascade of events in skeletal muscle initiates the process. During physical exercise, skeletal muscle burns significant amounts of carbohydrates and lipids, or even protein and nucleic acid, contributing to most of the total EE, and producing lots of metabolites at the same time. Owing to the high throughput metabolomic techniques, numerous metabolites have been screened and identified. These muscle-derived metabolites (myometabolites) facilitate crosstalk with other tissues including the brain, adipose tissue, liver, gut, and cardiovascular. They likely play a role in the health of these tissues and associated diseases, which coincided with the beneficial roles of exercise. These findings supply the molecular mechanisms underlying the beneficial effects of exercise. However, due to the complex diversity of metabolites, more work needs to be explored in-depth, especially the detrimental aspects of myometabolites. So that we can fully understand the physiological role of the metabolites, avoiding their negative effects on the body homeostasis as well as pathological treatment.

Hypoxanthine is the principal purine nucleobase involved in the purine salvage pathway, and also the intermediate of nucleotides metabolism (Farthing et al., 2015). During exercise or muscle contraction, ATP consumption is pronounced, which is accompanied by the Hx formation via AMP and IMP. The accumulated Hx is released from the muscle into blood and then transferred to other tissues. Although Hx can also be produced and released by other tissues, muscle is an important determinant of its level, because it constitutes such a high percentage of body mass and is highly metabolically active during contraction. Indeed, Hx in the muscle tissue is the most abundant compared with other tissues (Bone et al., 2015). Though its paracrine physiological roles in other tissues have been reported, the roles of Hx in the autocrine regulation of the muscles *per se* remain unknown. In the present study, we showed the Hx-induced muscular metabolism alteration and fatigue even in acute treatment, this rapid and dramatic effect is closely related to the role of Hx as a marker of exercise exhaustion or fatigue.

In this study, two doses of Hx (10 and 20 mg/g diet) had been conducted in the chronic Hx treatment. The lower dose of 10 mg/g Hx-treatment did not have significant effect on the growth and exercise ability of mice, while 20 mg/g Hx-treatment induced exercise weakness. The doses of metabolites in diet, 10 mg/g or 20 mg/g, are almost the maximal for the study about the physiological functions of metabolites as several research reported (Tian et al., 2020; Yuan et al., 2020). Higher doses might be toxic to animals and certainly change the nutrients concentration. Herein, 20 mg/g Hx-treatment caused muscle fatigue, but it had no effect on the growth and food intake of mice, and we did not observe any abnormal behavior of mice during the feeding experiment as well. In the acute Hx treatment, the effect on skeletal muscle was similar to that in chronic treatment, which appeared rapidly (30 min after i.p. injection), but without acute toxicity. These results exclude the toxicity of metabolite in Hx-induced muscle fatigue and further support the effect of Hx on muscle metabolism.

Uncoupling proteins, the mitochondrial anion carrier proteins, are located in the inner mitochondrial membrane and dissipate the proton electrochemical gradient generated during electron transport via the mitochondrial respiratory chain (Divakaruni and Brand, 2011; Bouillaud et al., 2016). UCPs uncouple mitochondrial respiration from ATP formation and the direct consequence of their activity is a decrease in the oxidative phosphorylation efficacy (Donadelli et al., 2014). UCP2 belongs to the UCP subfamily and is ubiquitously expressed in most cell types of different tissues (Donadelli et al., 2014). UCP2 expression variants were found to be associated with insulin sensitivity and diabetes mellitus (Jia et al., 2009; Diano and Horvath, 2012; Sreedhar and Zhao, 2017), obesity and metabolic syndrome (Li et al., 2003; Ricquier, 2007; Jia et al., 2009; Dinh et al., 2015; Sreedhar and Zhao, 2017), cardiac diseases (Brand and Esteves, 2005), immunity process (Emre et al., 2007; Emre and Nubel, 2010), tumorigenesis and cancer (Selimovic et al., 2008), aging (Brand and Esteves, 2005), and neurological diseases (Toda and Diano, 2014). Unlike its widely studied homolog UCP1, UCP2 protein level increases and decreases in a very short time, the half-life of UCP2 is short, approximately 30 min, which is about 30 h for UCP1 (Rousset et al., 2007). As expected, in the acute Hx-treated experiment, Hx worked in 30 min after i.p. injection, and we did not observe the decrease in muscle weight lifting and grip strength in 1 h after injection, which we did not show in this research. This highlights the importance of UCP2 in the cellular energy metabolism, which requires precise regulation. Recent advancements in the understanding of cellular glucose and lipid metabolism identified UCP2 as a critical regulator of cellular fuel utilization and the systemic glucose and lipid metabolism (Diano and Horvath, 2012). UCP2 overexpression enhanced glycolysis via activation of 6-phosphofructo-2-kinase/fructose-2,6-biphosphatase 2 (PFKFB2) during skin cell transformation (Sreedhar et al., 2017). On the contrary, Vozza et al. (2014) showed that *UCP2*-silenced human hepatocellular carcinoma (HepG2) cells, cultured in the presence of glucose, showed a higher inner mitochondrial membrane potential and ATP/ADP ratio associated with a lower lactate release. As expected, the mRNA and protein levels of UCP2 were significantly elevated in the skeletal muscle after Hx-treatment, which was accompanied with the increased muscular glycolysis and decreased ATP production. Then, knocked out of *UCP2* blocked the effects of Hx in skeletal muscle, which showed the indispensable role of UCP2 in Hx-induced muscle fatigue. Several studies have demonstrated the negative effect of ROS and oxidative stress in muscle function (Mailloux and Harper, 2012; Nautiyal et al., 2012; Deyu et al., 2018). In fact, UCP2 is a regulator of mitochondrial ROS, which is produced during oxidative phosphorylation by the electron transport chain. Through eliminating the accumulation of ROS, UCP2 could protect the cells against oxidative damage. Herein, we did not find a significant difference with Hx-treated mice compared to the control, but Hx induced increased mRNA expression of *Gss* and *Gpx4*, which mediate the glutathione reduction system. Therefore, we inferred that Hx-induced mitochondrial oxidative phosphorylation elevation caused ROS formation, which activated the UCP2 and other redox systems to neutralize the ROS. Furthermore, UCP2 contains purine nucleotide binding site in the structure (Vozza et al., 2014). So, as an intermediate metabolite of purine nucleotide, whether Hx could directly bind to UCP2 and affect its activity, or other regulators might mediate the crosstalk between Hx and UCP2, which need the in-depth investigation. Otherwise, UCP3 is another UCP that mainly exists in skeletal muscle. Unlike UCP2 activation, no alteration was found in the UCP3, which is consistent with divergent physiological functions of UCPs.

Overall, our study showed a new role of Hx in the skeletal muscle metabolism and function. Hx induced the elevation of UCP2-dependent mitochondrial uncoupling and blocked the respiratory chain from the synthesis of ATP. In addition, UCP2 caused excessive consumption of glycogen into lactate through enhanced glycolysis. These cases all together caused the skeletal muscle fatigue. Our results demonstrate a new perspective on the physiological role of myometabolites. This finding might be the potential pathological mechanism of muscle fatigue that requires further confirmation. On the other hand, it might be identified as a new strategy for the protection of the body from damage caused by excessive exercise.

## Data Availability Statement

The raw data supporting the conclusions of this article will be made available by the authors, without undue reservation.

## Ethics Statement

The animal study was reviewed and approved by The Instructive Notions with Respect to Caring for Laboratory Animals (Ministry of Science and Technology, Beijing, China).

## Author Contributions

QJ, HZ, and CY designed the project and experiments. CY and QJ wrote the manuscript. CY, ZM, FL, and CD performed the experiments. CY, ZM, and HZ collected, analyzed, and interpreted the data. XZ, PG, and GS provided the technical expertise and discussed the data. QJ, CY, HZ, LW, YY, CZ, and SW discussed, reviewed, and edited the manuscript. QJ and HZ supervised the work. All authors approved the final manuscript.

## Conflict of Interest

The authors declare that the research was conducted in the absence of any commercial or financial relationships that could be construed as a potential conflict of interest.

## Supplementary Material

Supplementary Figure 1 Locomotor activity (bean breaks) of mice after 4 weeks Hx treatment (*n* = 8 mice per group). Values are presented as means ± SEM.

Supplementary Figure 2 **(A–C)** Serum AST **(A)**, ALT **(B)**, and BUN **(C)** levels after acute Hx treatment (*n* = 8 mice per group). Values are presented as means ± SEM.

Supplementary Figure 3 **(A)** ROS level in the GAS muscle (*n* = 8 mice per group). **(B)** mRNA expression of *Gss* and *Gpx4* in the GAS muscle (*n* = 8 mice per group). β*-actin* was used as loading control in the qRT-PCR. Values are presented as means ± SEM; ^∗^*P* ≤ 0.05 according to non-paired Student’s *t*-test between individual groups.

Supplementary Figure 4 Body weight **(A)** and body composition **(B)** of AAV injection mice before acute Hx treatment (*n* = 8 mice per group). Values are presented as means ± SEM.
