# Supplementary figures and images for: Hypoxanthine Induces Muscular ATP Depletion and Fatigue via UCP2
Source: Front Physiol. 2021 Mar 3;12:647743. doi: 10.3389/fphys.2021.647743 (PMC7966526; doi:10.3389/fphys.2021.647743)

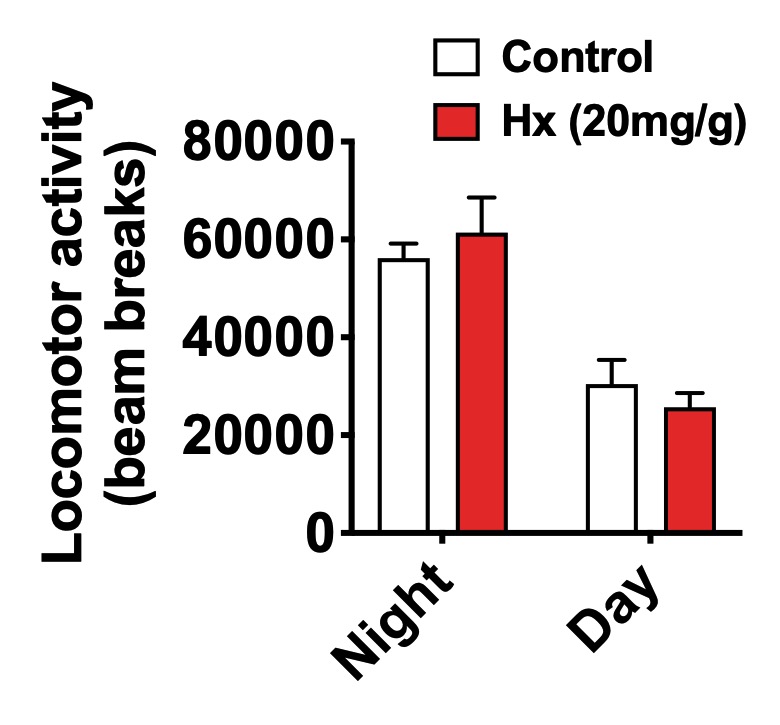

Supplement: Supplementary Figure 1 — Locomotor activity (bean breaks) of mice after 4 weeks Hx treatment (n = 8 mice per group). Values are presented as means ± SEM. [file Image_1.JPEG]

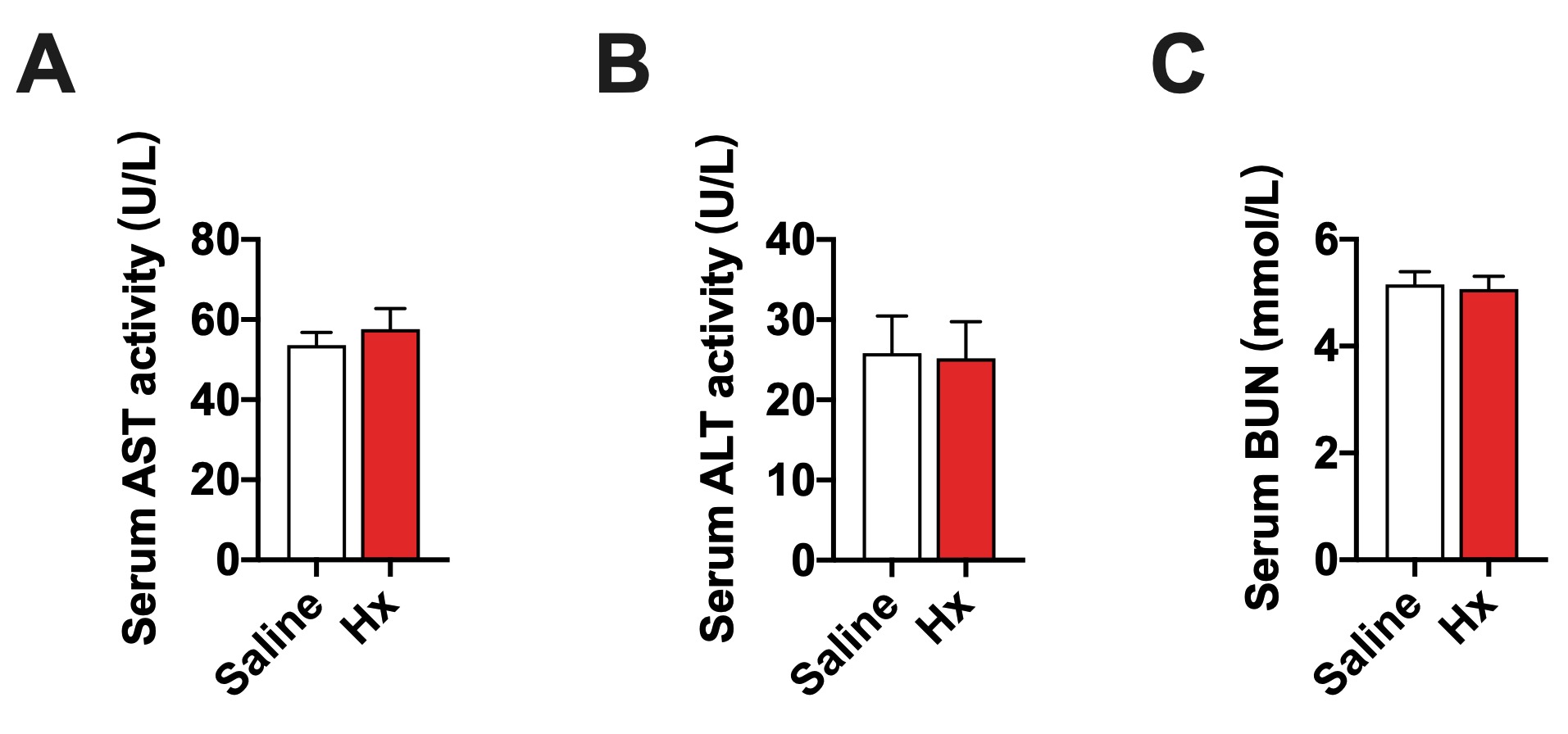

Supplement: Supplementary Figure 2 — (A–C) Serum AST (A), ALT (B), and BUN (C) levels after acute Hx treatment (n = 8 mice per group). Values are presented as means ± SEM. [file Image_2.JPEG]

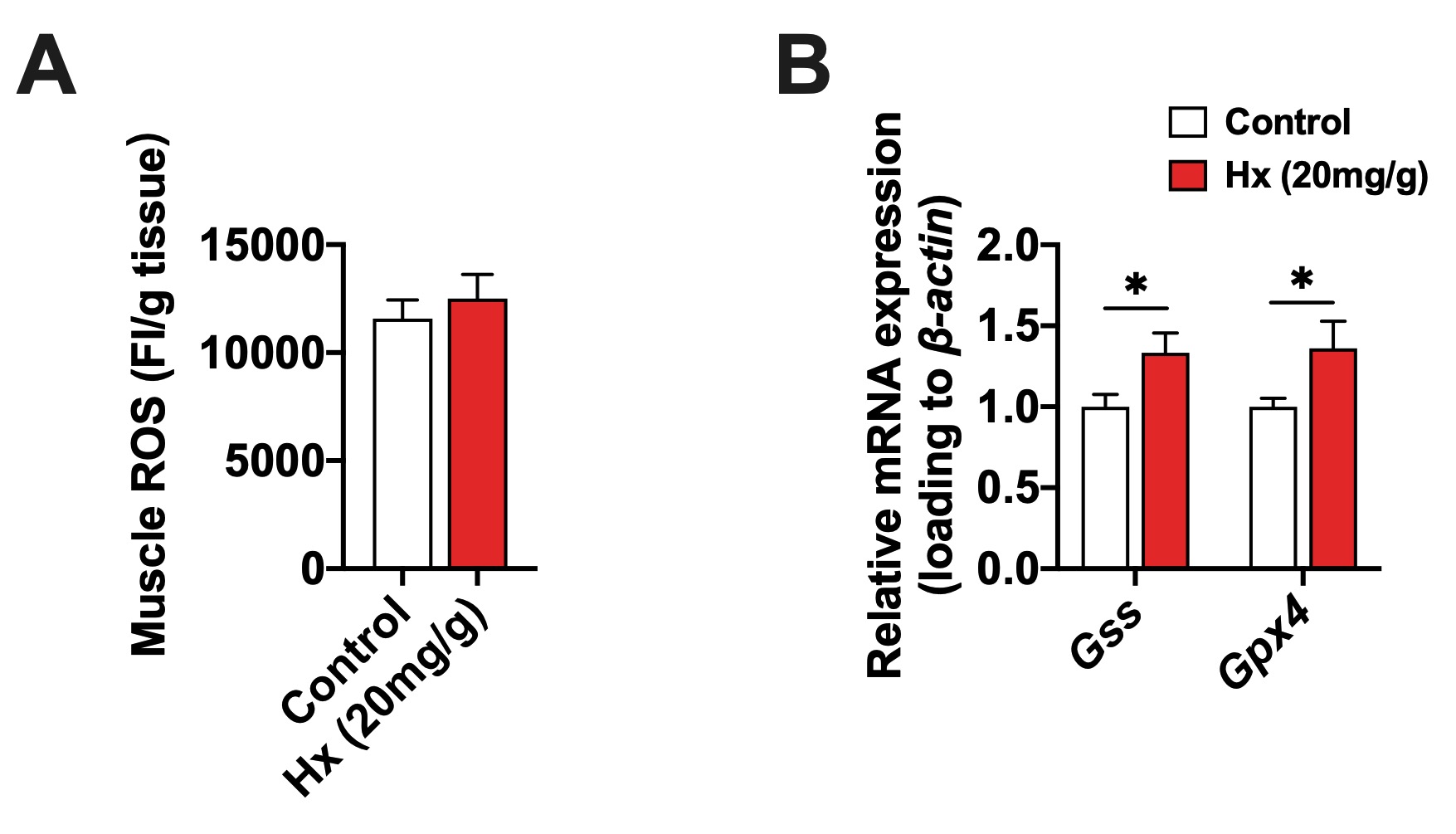

Supplement: Supplementary Figure 3 — (A) ROS level in the GAS muscle (n = 8 mice per group). (B) mRNA expression of Gss and Gpx4 in the GAS muscle (n = 8 mice per group). β-actin was used as loading control in the qRT-PCR. Values are presented as means ± SEM; ∗P ≤ 0.05 according to non-paired Student’s t-test between individual groups. [file Image_3.JPEG]

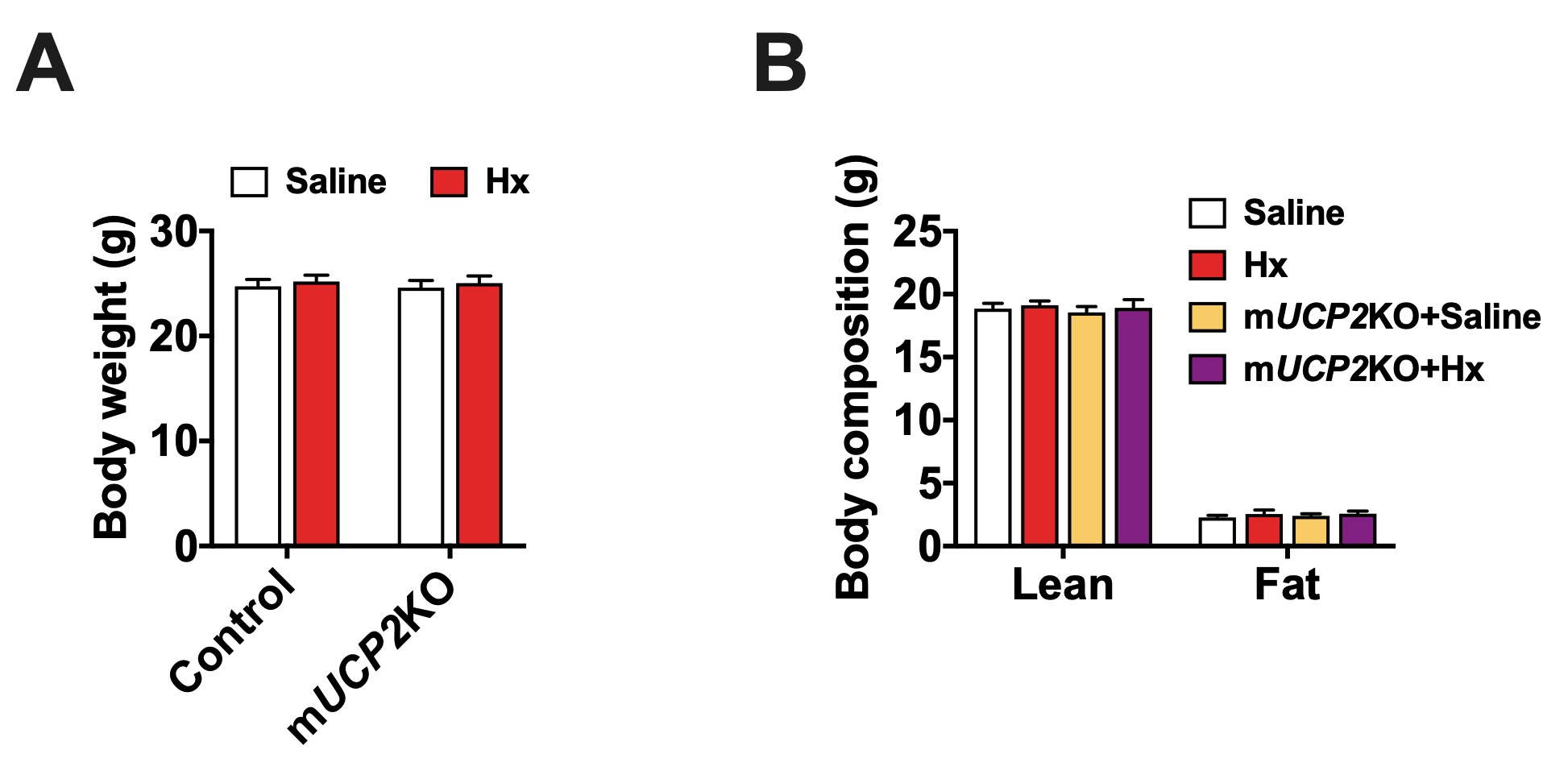

Supplement: Supplementary Figure 4 — Body weight (A) and body composition (B) of AAV injection mice before acute Hx treatment (n = 8 mice per group). Values are presented as means ± SEM. [file Image_4.JPEG]
